# Supplementary figures and images for: Integrated transcriptome and proteome analysis reveals the unique molecular features and nutritional components on the muscles in Chinese Taihe black-bone silky fowl chicken
Source: PLoS One. 2024 Mar 13;19(3):e0299385. doi: 10.1371/journal.pone.0299385 (PMC10936774; doi:10.1371/journal.pone.0299385)

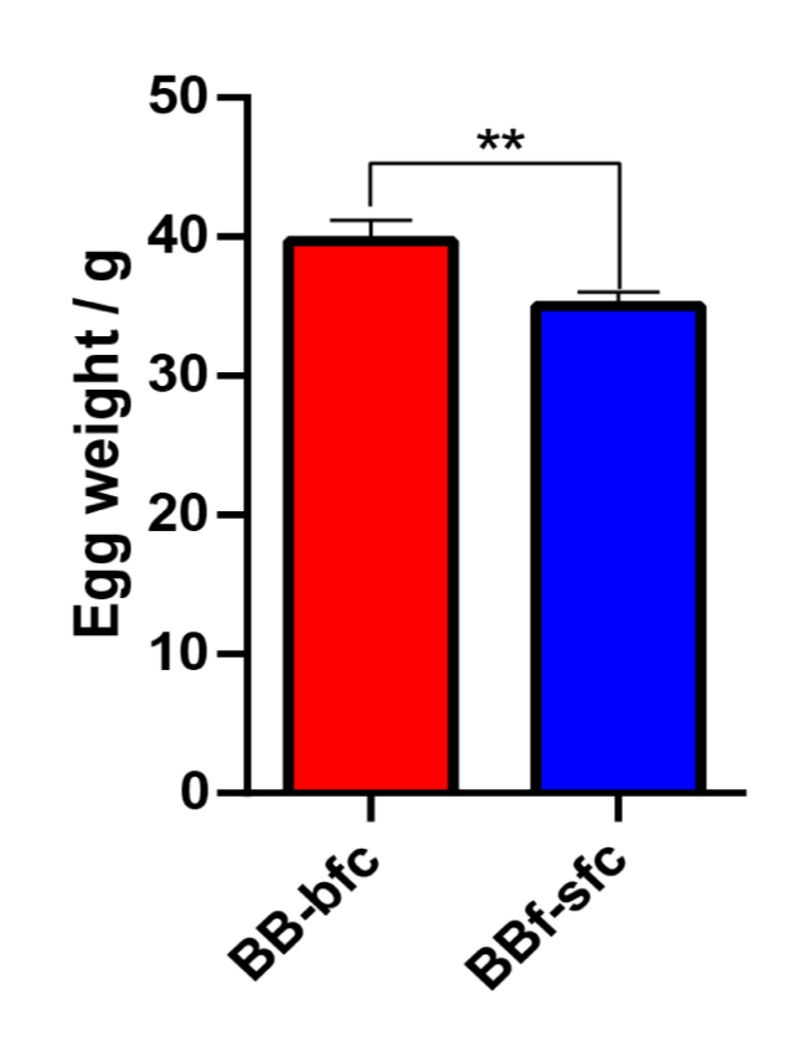

Supplement: S1 Fig — (TIF) [file pone.0299385.s001.tif]

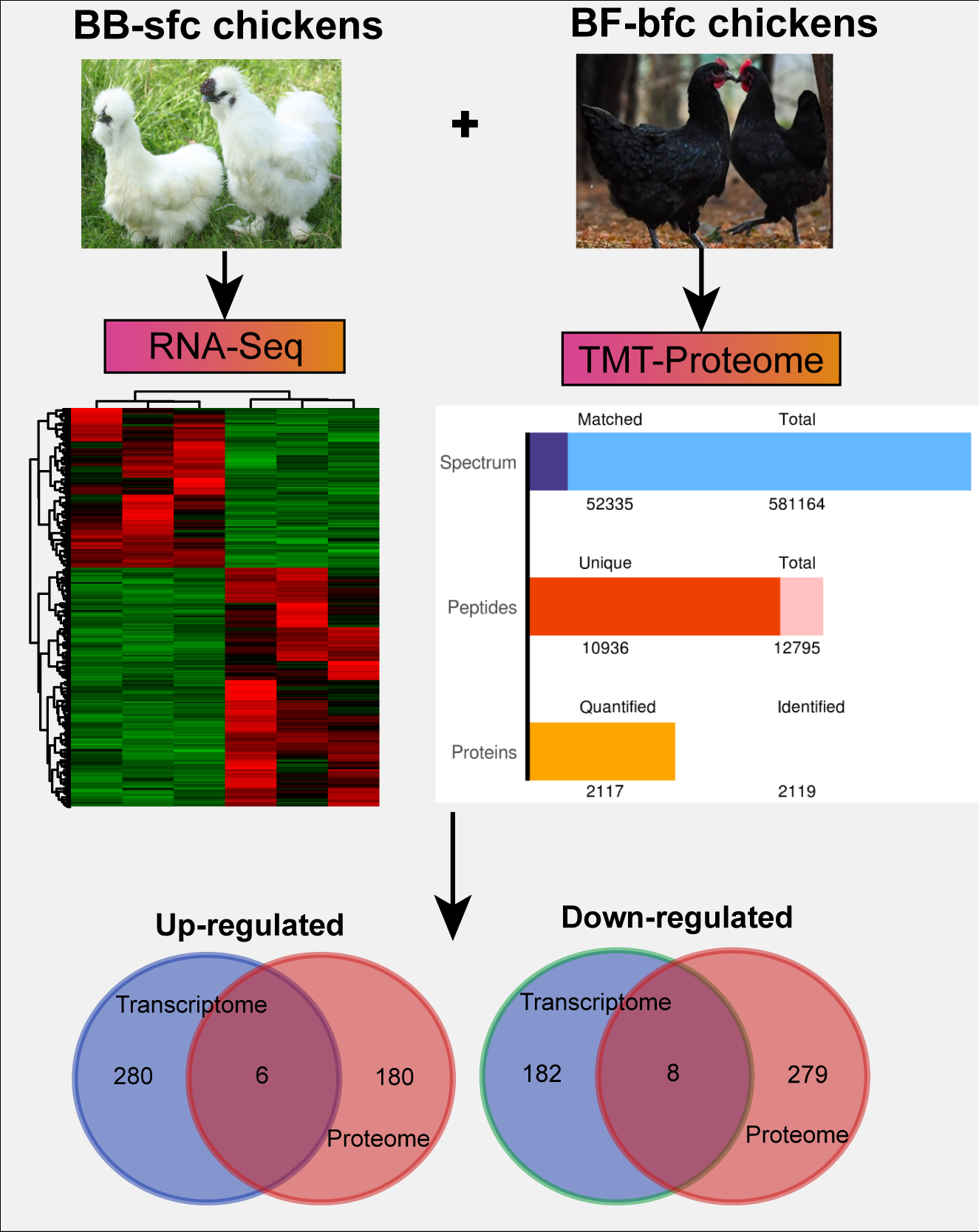

Supplement: S2 Fig — (TIF) [file pone.0299385.s002.tif]
