# Supplementary material for: Integrated transcriptome and proteome analysis reveals the unique molecular features and nutritional components on the muscles in Chinese Taihe black-bone silky fowl chicken
Source: PLoS One. 2024 Mar 13;19(3):e0299385. doi: 10.1371/journal.pone.0299385 (PMC10936774; doi:10.1371/journal.pone.0299385)
Supplement: Attachment — Submitted filename: Response letter.docx [file pone.0299385.s007.docx]

**Response Letter**

Dear editor and reviewers,

We are happy to send this revised version of the manuscript entitled “**Integrated transcriptome and proteome analysis reveals the unique molecular features and nutritional components on the muscles in Chinese Taihe black-bone silky fowl**”. We appreciate that our study has been found to be of interest, bringing novelty of relevance to the area of genetic breeding in poultry.

We take the opportunity to thank the editor and the reviewers for their time and expert appraisal of our study. We believe that the points raised by the reviewers have been adequately addressed in this revised version, as seen by the changes detailed and highlighted by the red color text in the R1 manuscript.

The detailed point-by-point responses are listed below.

Reviewer #1: Review of PONE-D-23-37042, Integrated transcriptome and proteome analysis reveals the unique nutritional composition on the muscles in Chinese Taihe Black-bone Silky Fowl (Gallus gallus domesticus Brisson) by Guanghua Xiong et al. The manuscript describes results of the integrated analysis of the transcriptome and proteome of muscles of two native Chinese chicken breeds with unique nutritional values. In itself such studies are interesting, even when the breeds themselves are of limited (commercial) value.
**Major comments**
1. The present manuscript lacks language clarity and should be revised. Usually, I provide a list of language suggestions, but here the list would have been 10 pages or so…… So, I suggest to contact a language editing company to improve the English presentation of the results.

**Answer：**Thank you for your nice suggestions. We have extensively revised and polished the manuscript through a professional language editing company, and all modified parts are marked in red in the manuscript

2. The Discussion section needs extensive rewriting, basically to the level of setting up an entire new Discussion section. Part of the results section need to be moved to the Discussion section, the results need to be removed from the Discussion section, and conclusions need to be drawn from the results after discussing them with the existing literature. Most of these points are either lacking from the present Discussion section, or the readability is such that I did not get it.
 **Answer：**Thank you for your good suggestions. We have rewritten the discussion section according to your suggestion and moved some of the results to the discussion section, while deleting the experimental results restatement in the discussion section.

**Minor comments**
Line (L)17: nutritional components: What do you mean? What the chicken eats? What the nutritional value is for consumers? Please be specific.

**Answer：**Thank you for your nice suggestions. Firstly, the nutritional components means the silky fowl chicken have higher nutritional substances in their muscles such as amino acids, trace elements, and unsaturated fatty acids. Secondly, the normal chicken diet mainly contains 60% corn, 20% soybean meal and 10% wheat bran. Thirdly, the nutritional value of anti-aging, anti-cancer, and immune enhancing functions for consumers. We have revised the manuscript in the abstract and method sections.
L18: Biomarkers for what?

**Answer：**Thanks. Biomarkers means the differentially expressed genes and proteins in Taihe silky fowl chickens, which may serve as distinguishing markers between Taihe silky fowl chicken and other breeds of chickens.
L19: Growth: general growth, or muscle development in specific?

**Answer：**Thanks. In this article, we specifically refer to muscle development.
L23: laid green-shelled eggs: only later it became known to me that this is possibly a breed, it is unclear here. Just add “chicken” or “breed” and the reader is not confused anymore.

**Answer：**Thanks for you good suggestions. We will uniformly name “Taihe silky fowl” as “Black-Bone silky fowl chicken (BB-sfc)”, and “Black Feathered chicken and laid green-shelled eggs” as “Black-Bone black-feathered chicken (BB-bfc)”.
L35: Overlapped: what do you mean by overlapped? transcriptome and proteome? What about the others? Are they false positives?

**Answer：**Thanks for you nice suggestion. “Overlapped“ means that these genes and proteins appeared simultaneously in the transcriptome and proteome analysis. Perhaps there are false positives in this, and we will conduct experimental confirmation on these genes and proteins in future research.
L62-64: Please rewrite sentence

**Answer：**Thanks for your reminder. We have rewritten this sentence.
Section 3.1 seems to contain no experimental results of this study. correct?

**Answer：**Thanks for your suggestions. Fig. 1A mainly aims to show the physiological differences between Taihe silk fowl chicken and local Black-feathered chicken, including differences in appearance and egg weight. Some data was provided in the supplementary materials. Fig. 1B mainly presents the main process transcriptome sequencing and proteome analysis.
L231-235: This is Materials ands Methods. Please delete here. If necessary, move to the Materials and Methods section.

**Answer：**Thanks for your suggestions. We have moved these sentences to the Materials and Methods section.
L264-267: Not in the Results section

**Answer：**Thanks for your suggestions. We have deleted these sentences.
L283-285: Move to the Discussion section.

**Answer：**Thanks for your reminder. We have moved it to the Discussion section.

L301: micro-organs? Do you mean organelles?

**Answer：**Thanks for your reminder. This word of “micro-organs” is indeed a bit ambiguous. We specifically refer to organelles. We have deleted this word.

L336-339: Move to the Discussion section.

**Answer：**Thanks for your reminder. We have moved it to the Discussion section.
L346-348: Move to the Discussion section.

**Answer：**Thanks for your reminder. We have moved it to the Discussion section.

L372-378: What is the meaning of this? It is so unclear to me that I cannot make a suggestion what to do with it.

**Answer：**Thanks for your suggestions. This sentence is indeed a bit ambiguous, and we have deleted it.
L398-400: Repetition of results is no discussion. What are the meanings of your results? Please discuss that.

**Answer：**Thanks for your suggestions. We have revised this section and discussed the meanings of our results.

References: no given names of authors

**Answer：**Thanks for your reminder. We have adopted the reference format specified by the PLOS ONE magazine.

Reviewer #2: General comments: The authors have put together an interesting data on the muscle of Chinese Taihe Black-bone Silky Fowl. However, there are some modifications to be made to this manuscript before is suitable for publication.
1. Line1-2: What is the unique nutritional composition of muscle in Chinese Taihe Black-bone Silky Fowl? I can not find that in the manuscript.

**Answer：**Thank you for your good suggestion. The Chinese Taihe Black-bone silky fowl chicken (BB-sfc) has high nutritional and medicinal value, rich in effective ingredients, and contains 18 amino acids higher than other chicken breeds^[1]^. According to previous literature reports, BB-sfc chicken is an alkaline food with high protein, low fat, and low sugar content, with an average crude protein content of 52.72% and crude fat only accounting for 24.17%, which the low fat ratio is comparable to soybeans and algae^[2]^. According to our RNA-Seq and proteome analysis, we identified 6 proteins (CBFB, POSTN, AGPAT2, CRP, METTL7A and MTPN) were up-regulated and 8 proteins (CYTB, AMPD1, CARNS1, CKMT2, PCCB, DUSP26, IDH3A and FN3K) were down-regulated in BB-sfc chickens. These identified proteins may be unique nutritional composition of muscle in Chinese Taihe Black-bone Silky Fowl.

[1] Liao X, Shi X, Hu H, Han X, Jiang K, Liu Y, et al. Comparative Metabolomics Analysis Reveals the Unique Nutritional Characteristics of Breed and Feed on Muscles in Chinese Taihe Black-Bone Silky Fowl. Metabolites. 2022;12(10).

[2] Zhou X, Liu L, Wang L, Liu T, Wu X. Proteomic study of Chinese black-bone silky fowl and the ring-necked pheasant egg white by iTRAQ technique. LWT. 2021;150:111936.

2. Line 23: What does ‘Black Feathered chicken and laid green-shelled eggs’ mean? Black Feathered chicken? laid green-shelled eggs?

**Answer：**Thank you for your good suggestion. We did not express this sentence clearly. Actually, there are many different breeds of Taihe black-bone chickens in China, among which the most typical is the black-bone silky fowl chicken (Namely BB-sfc). This breed of chicken has white feathers, black bones and meat, and lays eggs with red shells. Another breed is also a well-known local black-bone chicken, which we called the black-bone and black-feathered chicken (Namely BB-bfc). It has black feathers, black bones and meat, but the eggs it lays is green shells. The comparison pictures of the differences between these two breeds of chickens were shown as follows. We have made modifications to the corresponding sentences in the manuscript

3. Line 23: Why was Black Feathered chicken selected for transcriptome and proteome analysis? Which breed does Black Feathered chicken belong to?

**Answer:** Thank you for your question. The main objective of this article is to identify genetic diversity and differentially expressed genes and protein components in Taihe Black-Bone silky fowl chicken (BB-sfc) through proteomic and transcriptome analysis. The Black-Bone and black-feathered chicken (BB-bfc) is also a representative breed of Taihe black-bone chicken, so we chose it as a control with Taihe black-bone silky fowl chicken (BB-sfc).

4. Line 102: abdominal muscle tissues?

**Answer：**Thank you for your reminder. This statement is indeed a bit ambiguous. We are referring to “chicken breast meat”. We have made modifications to the relevant sentences in the revised manuscript.

5. Line 115-116: ‘all the chickens were randomly selected’ should be modified to ‘all the chickens were selected’

**Answer：**Thank you for your suggestion. I have made the modifications to this sentence in the revised manuscript.

6. Line 117: How were the chicken euthanized?

**Answer：**Thank you for your question. The chickens were euthanized by using a firm percussive blow to the head followed by manual or mechanical cervical dislocation. We have added these statements in the 2.1 Materials and Methods section.
 7. Line 201-202: Which statistical analyses in the study were performed using the SPSS 20.0 software?

**Answer：**Thank you for your question. The statistical analysis using the SPSS 20.0 software is egg weight comparison, including body weight, feed intake, egg production, and feed conversion ratio between two chicken breeds.

8. Line 214-215: where was the data on eggs obtained from?

**Answer：**The data on eggs obtained from the average of 40 eggs between the two breeds of chickens, which is approximately 36.2 ± 3.1 g (BB-sfc) vs. 39.5 ± 4.3 g (BB-bfc). This data was also shown in Supplementary Fig. S1.

9. Line 218: What does ‘data not shown’ mean? Is that your result?

**Answer：**During raising these two breeds of chickens, we recorded many growth indicators including body weight, feed intake, egg production and feed conversion ratio etc., and we have included these data in Supplementary Table 3. Meanwhile, we deleted the words of “data not shown” in the revised manuscript.

10. Line 221-224: Why did proteomic quantitative analysis was performed using four biological replicates of muscle samples but RNA-Seq three biological replicates?

**Answer：**Thank you for nice suggestion. Based on previous research experience, transcriptome sequencing only requires three biological replicates per sample, but individual differences in samples of TMT-based proteome sequencing are larger than RNA-Seq. Therefore, in order to ensure the reliability of the results, we increased the number of biological replicates to four per sample in proteomic quantitative analysis.
11. Which part of result does the Figure 7 belong to? I did not find Figure 7 in the results and and legend of Figure 7 of manuscript.

**Answer：**Thank you for your reminder. Figure 7 is the Grapgh Abstract (Striking image) of the entire article, which should have appeared separately on the online version of the paper's webpage, so it was not included in the manuscript of the main text.
12. Please upload your original sequencing data.

**Answer：**Thank you for your reminder. The transcriptomics datasets generated during the current study are available in the NCBI SRA (PRJNA1051302). The mass spectrometry proteomics data have been deposited to the ProteomeXchange Consortium ([http://proteomecentral.proteomexchange.org](http://proteomecentral.proteomexchange.org/)) via the iProX (Integrated proteome resources, [http://www.iprox.org/) partner](http://www.iprox.org/)%20partner) repository with the dataset identifier PXD047777. We have revised this information in the section of “data availability statement”.

Reviewer #3: The manuscript is written clearly with the exception of some few areas that need to be addressed as follows:
1. there are several grammatical errors- L40-41, 62, 68, and 112.

**Answer：**Thank you for your suggestions. We have made the following modifications to these sentences.

**L40-L41.** “Taken together, this research provides new insights into the molecular mechanism of antioxidant capacity in chicken muscles, and our study will laid the scientific basis for the improvement of the growth performance in Taihe black-bone silky fowl.”

**Reviesed to** “Taken together, our studies provides a large database of gene expression and protein information of Taihe Black-Bone silky fowl chicken in muscles, which will provide a theoretical basis for fully exploring the unique economic value of Taihe BB-sfc chickens in the future.”

**L62:** “Due to traditional breeding require long-term selection and usually time-consuming, molecular breeding based on high-throughput sequencing can greatly improve breeding efficiency and shorten breeding cycles”

**Revised to** “Since conventional breeding requires long-term selection and generally takes a long time, the modern molecular genetic breeding using on high-throughput sequencing can significantly improve breeding efficiency and shorten the breeding cycle.”

**L68: “**which can be used to study gene expression and gene function prediction”

**Revised to** “which can be used to study the differentially expressed genes and regulated signaling pathways”.

**L112: “**the chickens of Black-feathered chicken and laid green-shelled eggs (BF-gsc)”

**Revised to** “Black-Bone and black-feathered chicken (BB-bfc)”
2. avoid repeating methods in results-L210-212, 231-233, and 367-374.

**Answer：**Thank you for your suggestions. We have deleted the corresponding sentences in L210-212, 231-233, and 367-374.
