# Supplementary material for: Integrated transcriptome and proteome analysis reveals the unique molecular features and nutritional components on the muscles in Chinese Taihe black-bone silky fowl chicken
Source: PLoS One. 2024 Mar 13;19(3):e0299385. doi: 10.1371/journal.pone.0299385 (PMC10936774; doi:10.1371/journal.pone.0299385)
Supplement: Attachment — Submitted filename: Response letter R3.docx [file pone.0299385.s008.docx]

Reviewer #2: The authors have put together an interesting data on the muscle of Chinese Taihe Black-bone Silky Fowl.
Minor comment:
1.Line140-141: |fold changes| or |log2(fold change)| ?

**Answer:** Thank you for your nice reminding. We have revised it to |log2(fold change)|.

Reviewer #3: The manuscript has been revised adequately. All the issues raised have been adequately addresses including depositing of data in the NCBI.

**Answer:** Thank you for your positive comments.

Reviewer #4: In your research, you performed RNA-Seq, transcriptome, and proteome analyses using Black-Bone silky fowl chicken.　You identified genes from these analyses that were associated with improving the antioxidant status of muscles.　However, I have some suggestions.
1. Please make the abstract section a little more concise.

**Answer:** Thank you for your good suggestions. We have reduced the abstract section from 331 words to 223 words.

2. Please include the results of the RNA sample quality test in the methods or supplemental section to prove the reliability of the data.　For example, nanodrop and bioanalyzer tests.

**Answer:** Thank you for your nice suggestion. We have added the RNA sample quality in the supplemental Table S4.

3. Please add a conclusions section with your results included.

**Answer:** Thank you for your nice suggestion. We have added a conclusion section after tha discussion section.

4. Please revise the discussion section to clearly show the relevance of your results. Currently, it is difficult to understand the relationship between your results and references to previous studies.

**Answer:** Thank you for your nice suggestion. We have made significant modifications to the discussion section, making our research results more relevant to previous studies.

5. You note the “unique economic value of Taihe BB-sfc chickens in the future” Please explain in more detail. Why and how is it useful?

**Answer:** Thank you for your nice suggestion. The Chinese Taihe silky fowl chicken is a national product of geographical indication that known for its top ten characteristics, high nutritional and medicinal value. The chicken of Taihe Black-bone silky fowl is fresh and tender in meat with high protein and low fat, which the average crude protein content of 52.72% and crude fat only accounting for 24.17%. It is understood that Taihe silky fowl chicken is the main source of income for local farmers, but the main obstacle to promoting Taihe black-bone chicken is that its unique nutritional composition is not yet clear. Therefore, it is necessary to clarify the differences in muscle composition between Taihe silky fowl chicken and other black-bone chickens through transcriptome and proteome sequencing methods.
